# Up-Regulated Expression of *AOS-LOXa* and Increased Eicosanoid Synthesis in Response to Coral Wounding

**DOI:** 10.1371/journal.pone.0089215

**Published:** 2014-02-14

**Authors:** Helike Lõhelaid, Tarvi Teder, Kadri Tõldsepp, Merrick Ekins, Nigulas Samel

**Affiliations:** 1 Department of Chemistry, Tallinn University of Technology, Tallinn, Estonia; 2 Sessile Marine Invertebrates, Queensland Museum, Brisbane, Queensland, Australia; Mount Allison University, Canada

## Abstract

In octocorals, a catalase–like allene oxide synthase (AOS) and an 8*R*-lipoxygenase (LOX) gene are fused together encoding for a single AOS-LOX fusion protein. Although the AOS-LOX pathway is central to the arachidonate metabolism in corals, its biological function in coral homeostasis is unclear. Using an acute incision wound model in the soft coral *Capnella imbricata*, we here test whether LOX pathway, similar to its role in plants, can contribute to the coral damage response and regeneration. Analysis of metabolites formed from exogenous arachidonate before and after fixed time intervals following wounding indicated a significant increase in AOS-LOX activity in response to mechanical injury. Two AOS-LOX isoforms, *AOS-LOXa* and *AOS-LOXb,* were cloned and expressed in bacterial expression system as active fusion proteins. Transcription levels of corresponding genes were measured in normal and stressed coral by qPCR. After wounding, *AOS-LOXa* was markedly up-regulated in both, the tissue adjacent to the incision and distal parts of a coral colony (with the maximum reached at 1 h and 6 h post wounding, respectively), while *AOS-LOXb* was stable. According to mRNA expression analysis, combined with detection of eicosanoid product formation for the first time, the *AOS-LOX* was identified as an early stress response gene which is induced by mechanical injury in coral.

## Introduction

Octocorals and hexacorals are affected by natural and human-induced stress factors [1]. Biological and chemical alterations, including changes in temperature, sedimentation, water conditions and biotic alterations all represent challenges to octocorals survival. Mechanical injury is also a major challenge to coral integrity and viability [2]. It is likely that each type of stress leads to an adaptation or repair response, aiming to re-establish homeostasis and survival [3].

For example, altered temperature (such as heat shock) leads to changes in coral heat stress responsive genes and elevated calcium levels [4]. It also results in elevated levels of heat shock proteins, reactive oxygen species, Ca^2+^ signaling and protein synthesis [5], [6]. The identification of indicator pathways is relevant for the monitoring and prediction of environmental stress conditions in coral [7]. While coral response to stress has been well studied in reef-building corals (hexacorals) [4]–[7], the stress-response of soft corals (octocorals) remains largely elusive [8].

In both vertebrates and invertebrates, similar phases of wound healing (1- inflammation, 2- proliferation, and 3- matrix rebuilding and remodeling) have been described [9], [10]. However, the coral wound response and wound-related stress have received little attention [11], most research has concentrated on the response at the tissue level [12]–[16].

In vertebrates and plants, oxylipins are important stress mediators. In mammals, eicosanoids (hydroperoxyeicosatetraenoic acids (HpETEs), leukotrienes, thromboxanes and prostaglandins) result from the conversion of arachidonic acid (AA) by lipoxygenase (LOX) and cyclooxygenase (COX) [17], [18]. Due to their labile nature, these messengers act locally, in an auto- or paracrine manner, as part of inflammatory responses by immune cell activation during infection or anaphylaxis [19], [20]. In plants, the conversion of α-linolenic acid by the LOX and allene oxide synthase (AOS) pathway results in 12-oxo-phytodienoic acid and jasmonic acid (JA), which regulate the expression of defense genes [21]–[24].

Soft corals expresses multiple eicosanoid biosynthesis pathways, including COX, LOX and allene oxide synthase- lipoxygenase (AOS-LOX) enzymes [25]–[32]. The unique AOS-LOX fusion protein catalyzes the formation of unstable allene oxide from AA via 8*R*-HpETE, a pathway common among octocorals [32]. Bioinformatics also indicate, that catalase like *AOS-LOX* is present in all cnidarian lineages (*Hydra, Acropora, Nematostella*) [33]. As related species often share similar metabolic routes, the data on biological role of eicosanoids in soft coral may be attributed to other *Cnidarian* lineages.

Whereas the spectrum of metabolites is largely known, the functional significance of AOS and LOX pathways in coral homeostasis and regeneration remains elusive. The current literature of coral eicosanoids contains data on the identification of naturally occurring compounds [27], [34]–[36], the elucidation of metabolic pathways involved in their synthesis [26], [28], [29], [37], [38] and the effects of lipid extracts or isolated compounds on other systems [39]. To date, only the role of prostaglandins in the chemical defense of the coral *Plexaura homomalla* has been revealed [40]–[43]. There is no data available about the function of eicosanoids produced via the AOS-LOX pathway in corals.

In the current study, using the model of *Capnella imbricata,* which is easily propagated and farmed in a laboratory marine aquarium, we address gene expression and eicosanoid synthesis through the AOS-LOX pathway in response to acute incision wounding of coral.

## Materials and Methods

### Coral Samples

Colonies of soft coral *C. imbricata* (*Cnidaria, Anthozoa, Octocorallia, Alcyonacea, Nephtheidae*) were purchased from a commercial source (Estonia), identified at Queensland Museum (specimen No: QM G317136), cultivated and propagated in a closed-circuit marine aquarium in the Department of Chemistry at Tallinn University of Technology at an ambient seawater temperature of 23±0.5°C, salinity 31 ppt, periodic day-night cycle (12 h–12 h) and 20% of biweekly water exchange.

### Activity Assay

In a standard assay, the coral tissue (0.33 g mL^−1^) was homogenized (Tissue Tearor, set 5) in 50 mM Tris-HCl pH 8.0 buffer, containing 0.5 mM phenylmethylsulfonyl fluoride (PMSF) on ice. Immediately, an aliquot of homogenate (6.6 mg) was incubated with 50 µM [1-^14^C] AA (GE Healthcare) in 1 mL (final volume) 50 mM Tris-HCl, 100 mM NaCl and 1 mM CaCl_2_ pH 8.0 at room temperature, with constant stirring for 5 min. Incubation in the presence of a mild reducing agent (0.5 mM SnCl_2_) was conducted in parallel. Reactions were terminated with SnCl_2_ (10 mM) and, after acidification with HCl to pH 4.0 the products were extracted with ethyl acetate. The extract was dried over Na_2_SO_4_, evaporated to dryness and re-dissolved in methanol: water (4∶1) for instant product analysis by reverse phase-high performance liquid chromatography (RP-HPLC).

### RP-HPLC

Samples were analyzed by RP-HPLC, using a Zorbax Eclipse XDB-C_18_ column (5 µm, 4.6×150 mm), thermostat 35°C, run on an Agilent 1200 Series HPLC system, connected to a diode array detector (UV detection at 206 nm, 236 nm and 270 nm), followed by a 500TR Series Flow Scintillation Analyzer (Packard Bioscience) or Agilent 6540 UHD Accurate Quadrupole time of flight - MS/MS with Agilent Jet Stream ESI source. The HPLC was carried out with a solvent system of acetonitrile (ACN)/water/formic acid (98.9%/1.0%/0.1% v/v/v)(A) and water/formic acid (99.9/0.1% v/v)(B), 0–8 min isocratic (35% A:65% B), 9–17 min gradient to 100% A, 18–30 min 100% A at a flow rate of 1 mL min^−1^. Mass spectra were acquired over a mass range of m/z 100–400 in a negative ion detection mode. Extracted ion current (EIC) was used for sensitive and specific detection of stable end products. The data acquisition was performed by ±0.1 m/z units centered on each selected ion.

### Extraction of Total RNA and Synthesis of cDNA

#### For homology based RT-PCR

Total RNA was extracted from coral tissue using the phenol-chloroform extraction method [44]. First strand cDNA was synthesized from 20 µg of total RNA, using an oligo(dT)-adapter primer [ATGAATTCGGTACCCGGGATCC(T)_17_] for priming and M-MLV reverse transcriptase (Promega) according to the manufacturer’s protocol.

#### For real-time quantitative PCR (qPCR)

100±2 mg of coral tissue was homogenized (IKA T18 basic ULTRA TURRAX) in a QIAzol Lysis Reagent. RNA was isolated using an RNeasy Lipid Tissue Mini Kit (Qiagen) according to the manufacturer’s instructions. Isolated RNA was quantified with NanoDrop-3000 (Thermo Scientific), and the integrity was constantly confirmed by electrophoresis on 1% formaldehyde agarose gels. 1 µg of total RNA was treated with DNase I and used as a template in cDNA synthesis (QuantiTect Reverse Transcription Kit, Qiagen) with oligo(dT)primer in a total reaction volume of 20 µL. Negative controls without reverse transcriptase were included to test for genomic DNA contamination and the efficiency of cDNA synthesis.

### Homology Based RT-PCR

The upstream degenerative primers were based on the conserved regions of coral AOS-LOX sequences HEFF and HPW (located on the AOS domain), and the downstream degenerative primers were based on QIQ and AGT (located on the AOS and LOX part of the AOS-LOX sequence, respectively). The first round PCR was run using 1 µL of first strand cDNA and the Expand Long Template PCR System with buffer 3 (Roche Diagnostics), 0.2 mM of each dNTP, and 0.3 µM primers (Table 1A). The PCR program was 1 cycle at 94°C for 2 min; 10 cycles at 93°C for 30 s, 52°C for 45 s, 68°C for 3 min; 20 cycles at 93°C for 30 s, 55°C for 45 s; 68°C for 3 min and 20 s for each cycle, and 68°C for 10 min. The half-nested second round PCR was run using 1 µL of 10 times diluted first round PCR reaction according to the same protocol. HEFF/QIQ; HEFF/AGT and HPW/AGT resulted in expected amplicon sizes (accordingly 586 bp, 981 bp and 300 bp). The PCR products were cloned (pGEM-T Easy Vector Systems, Promega) and sequenced (Agowa, Germany). Two different sequences homologous to coral *AOS-LOX*s were confirmed by the public BLAST platform at NCBI. Sequence alignments were created using the MegAlign program (DNAStar, Lasergene) with ClustalW. The 5′- and 3′-ends were extended using 5′-3′ RACE-PCR methodology (Promega), according to the manufacturer’s instructions and sequence specific primers (Table 1B). The open reading frames coding full length *AOS-LOXs* were PCR amplified with specific primers (Table 1B) and Phusion High Fidelity DNA polymerase (Thermo Scientific). The PCR products were cloned, sequenced and submitted to a database (GenBank accession numbers: KF000373 and KF000374).

**Table 1:** List of primers: (a) degenerative primers used for isolation of the target genes, (b) primers used for 5′-3′ RACE and (c) qPCR primers used for gene expression analysis.

*(a) Degenerative primers*			
HEFF-up	CCTAAGTTYCCNGARCAYGARTTYTT		
HPW-up	TGGGATAARGARACNCAYCCNTGG		
QIQ-down	CTAGCYTCRTGDATYTGDATYTG		
WDK-down	CCATGGRTGNGTYTCYTTRTCCCA		
AGT-down	GTAATAGTNGCRTCNGTNCCNGC		
***(b) Specific primers***			
**5′ RACE**			
FPV-down	TTGCATGACGTAATCTTACAGG		
FWHT-down	AGTCTTCCAAGCTCGAAGTGTGC		
FWNT-down	AGTCTTCAAAGCTTGAGGTGTTCC		
GSD-down	AGAACGATCTAGCATCAGACCC		
KYPD-down	CAGCACCTGCATCATCTGGATAC		
LKLL-down	CCCTGCATCATCCAGTAGTTTAAG		
**3′ RACE**			
DYHL-up	AAAGTTAACCTGCAAGACTATCATC		
EESG-up	ATATCAAAGAGGAAGAAGAGAGTGG		
ESLG-up	TCAACCAGCCGGAATCATTAGG		
FAVS-up	ACAACTGAATCATTTGCTGTGTCG		
FSRY-up	AAATATTTGGACATTCAGTCGTTATG		
KTHG-up	AATATCAAGGCTAAAACACACGG		
LGDT-up	CTGGATACTTGGTGATACGCC		
QNAL-up	TTCCAACAGGACAGAACGCAC		
RSRH-up	AACGTCTGGATTCGTAGTCGTCATC		
YKWI-up	TGTGGCAGTGTACAAATGGATCC		
AWED-up	AGCGCTACAGCTTGGGAGG		
ERIP-up	TTCTTCCTGAGCGTATTCCC		
***AOS-LOXa*** ** ORF**			
ALA-up	ATCGGATCCATGACTTGGAAAAATTTTGGA		
ALA-down	GTTCGGATCCCCGGGACATTAGATAGCAGTTCC		
***AOS-LOXb*** ** ORF**			
ALB-up	ATTGCTAGCATGGTTTGGAAAAATTTTGGTTACG		
ALB-down	CAGCTAGCCTAGATTGCAGTTCCG		
***(c) Specific qPCR primers***			
***Gene***	***Primer***	***Sequence***	***Amplicon size***
*AOS-LOXa*	FSRY-up	AATATTTGGACATTCAGTCGTTATG	100 bp
	LKKG-down	CGATAGTTTACTGGGCCTTTCTTC	
*AOS-LOXb*	KLLD-up	CGTCATGCAAATCTTAAACTACTGG	180 bp
	VSSS-down	AGACTCTCCTGCACTTGATGATAC	
*β-actin*	HETC-up	TGTGGCATCCATGAGACCTG	95 bp
	TVLS-down	AGACAGCACTGTGTTGGCATAC	

Up - forward primer, down - reverse primer; all sequences are presented in the 5′ to 3′ direction.

### Bacterial Expression and Activity

The ORF of *AOS-LOXa* and *AOS-LOXb* fusion proteins were PCR amplified with specific primers (Table 1B), cloned into *Nhe*I or *Bam*HI restriction site (respectively) of pET11a expression vector (Stratagene) and expressed in *Escherichia coli* BL21(DE3)RP cells (Novagen) at 10°C as previously described [32]. Bacterial extracts expressing the fusion protein were separated on 10% SDS-PAGE (Coomassie Blue stained). The fusion proteins (corresponding to the 1 mL sonicated cell culture) were incubated with 50 µM [1-^14^C] AA in 1 mL, and analyzed as described above.

### Design of Wounding Experiments

#### Repeated wounding (one colony)

**Figure 1:**
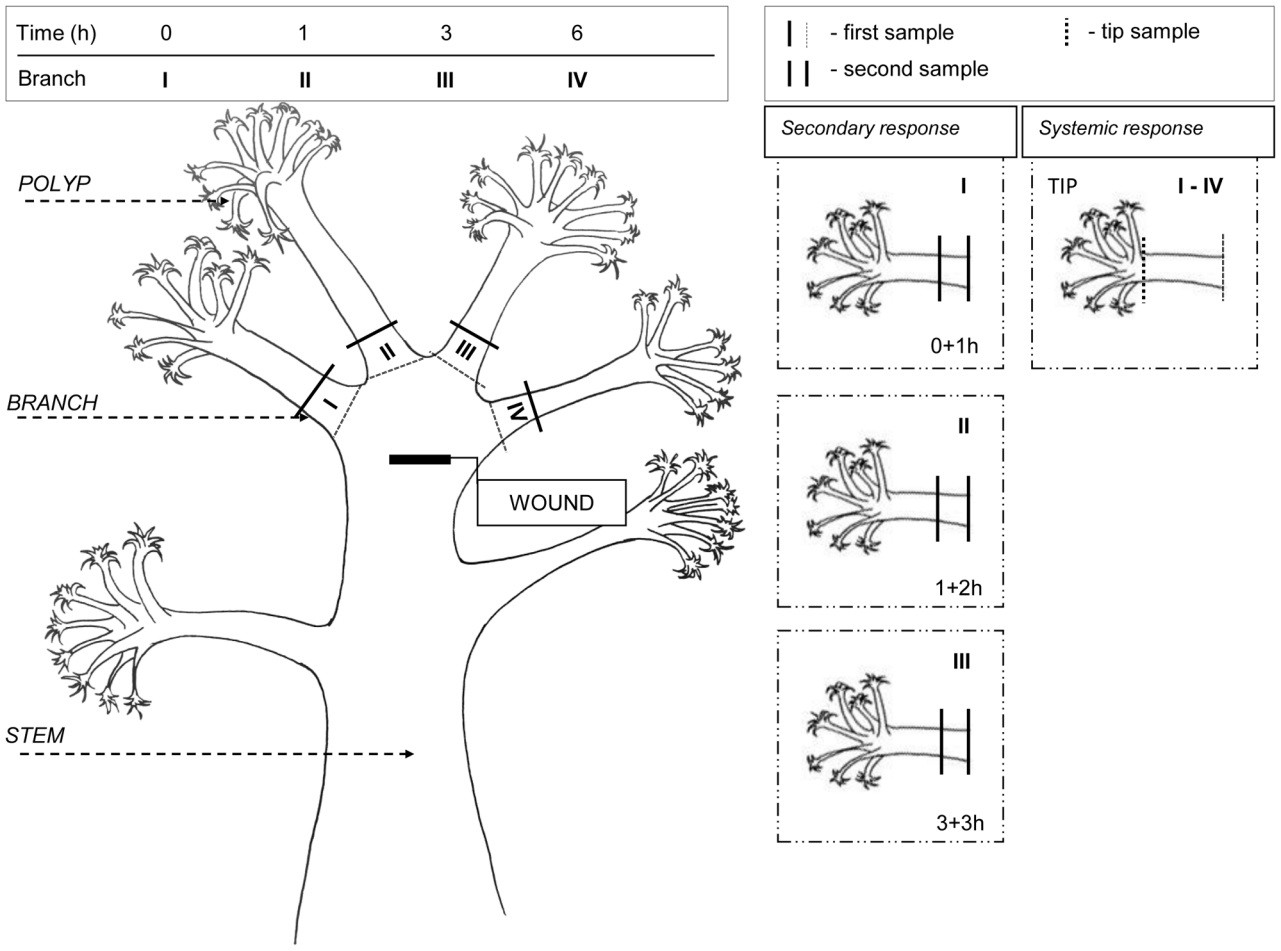
Experimental design of wounding stress. The location and time of sampling shown on a coral colony.

A coral colony was injured at the stem by a cut (0.5×5 mm), and 7–8 cm branches of the same colony were cut away at different times: zero (I branch), 1 h (II branch), 3 h (III branch) and 6 h (IV branch) (Fig. 1). A tissue sample (adjacent to the cutting edge) was taken, weighted, homogenized, and RNA was extracted. The remaining branch was additionally incubated until the next time point (1 h), resulting in a secondary incubation time of (0+1 h) (Fig. 1). The same scheme was repeated with branches II and III, and they were additionally incubated (for 2 and 3 h, respectively), marked as 1+2 h and 3+3 h (Fig. 1).

For the detection of a systemic response, transcript levels were estimated for the distal tips of the removed branches at given times (1 h, 3 h and 6 h), and they were compared to that of the time of wounding (control).

#### Single wounding (separate colonies)

To obtain genetically identical coral, an adult coral colony was cut into six equal fragments and grown for 4 months to reduce the influence of fragmentation. A branch was removed from the stems of three coral colonies in parallel, serving the wounding event and used for the detection of the normal transcript level for each colony (control). The second sample adjacent to the wounding site was taken from the first to the third coral colony at 1 h, 3 h and 6 h post wounding.

### qPCR and Gene Expression Analysis

*β-actin* was one of the three most stable genes identified in the cnidarian case study [45]. Corresponding sequence from *C. imbricata* was cloned, sequenced and used as the reference gene. Sequence specific quantitative real-time PCR primers (Table 1C) were designed on the *β-actin*, *AOS-LOXa* and *AOS-LOXb* genes by using PrimerSelect software (DNASTAR, Lasergene). Test-PCRs confirmed the specific amplification of the desired amplicons (95–180 bp). All sequenced qPCR products matched the expected product identities. First strand cDNA aliquots (1 µL) of each sample served as templates for a quantitative PCR reaction (total volume 10 µL) containing sequence-specific primers (500 nM) and a master mix (QuantiTect SYBR Green PCR Kit, Qiagen). Thermal cycling was performed on a *LightCycler* 480 Real-Time PCR system (Roche), under the following conditions: 95°C for 5 min, followed by 40 cycles of 95°C for 10 s, 55°C for 20 s and 72°C for 20 s. To confirm the amplification of a single PCR product, a melting curve analysis (from 52°C to 95°C) was carried out after the end of the amplification cycle. All qPCR reactions were performed in triplicate, and negative controls consisting of un-transcribed RNA (no RT control) were performed for each RNA extraction. The normal expression levels were estimated in the “stem” and “branch” tissue of three different coral colonies (n = 3). The initial and single wound response data are the means of values obtained for the two independent biological replicates (n = 2) and the secondary and systemic response data are the means of three replicates (n = 3).

#### Statistical analysis

The expression ratio between the sample and control was determined using the comparative C_t_ method [46] by program REST - Relative Expression Software Tool-Multiple Condition Solver (REST-MCS) [47] (LightCycler Relative Quantification Software, Qiagen) with 2000 iterations. The results are presented as the mean ± standard error (SE).

## Results

**Figure 2:**
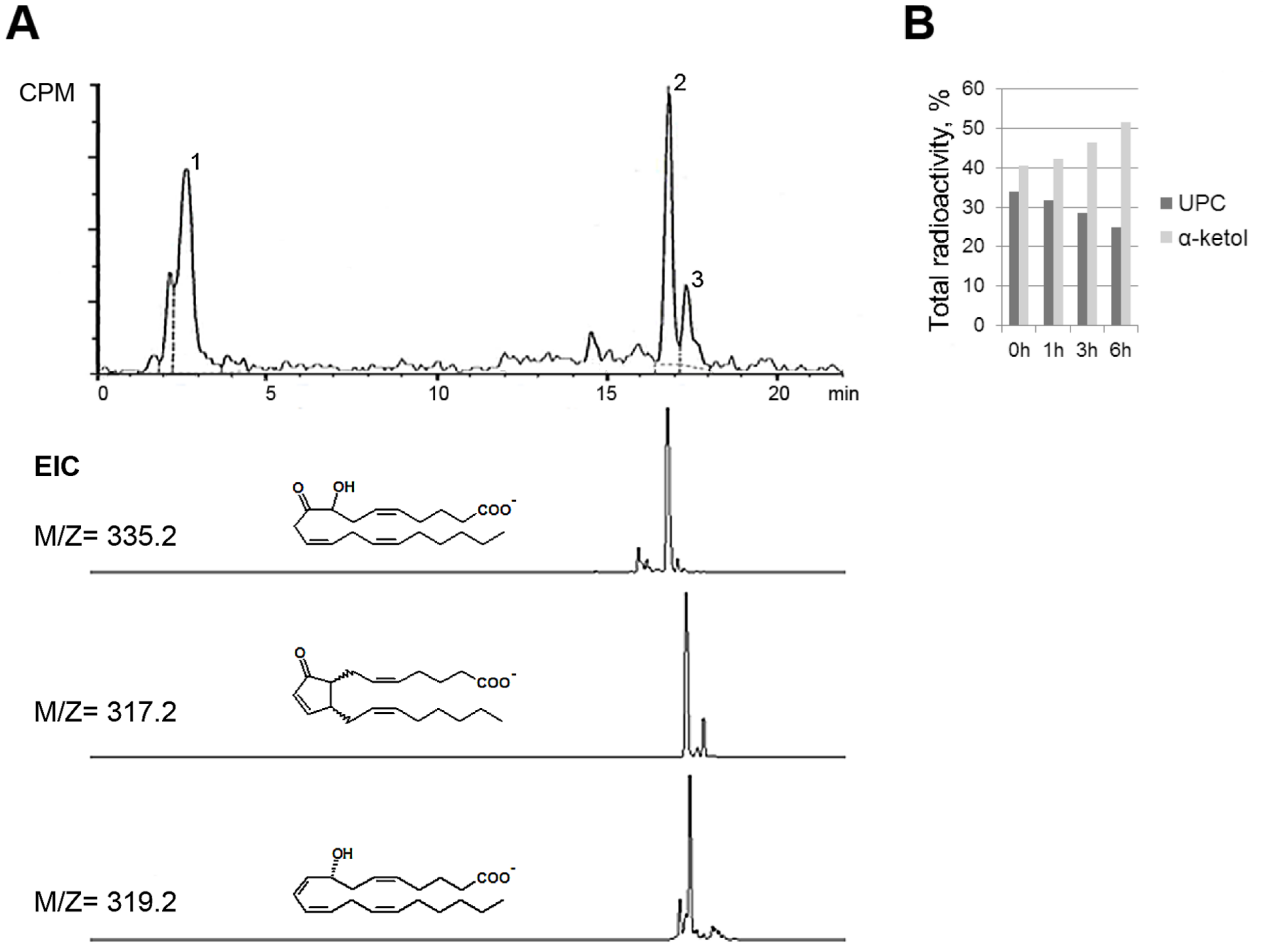
RP-HPLC analysis of incubation products of C. *imbricata* tissue homogenate. A) Radio chromatogram of the products formed from [1^−14^C] AA by coral homogenate, extracted ion current (EIC) corresponding to α-ketol ([M^−^] = 335.2, peak 2), cyclopentenone ([M^−^] = 317.2, peak 3) and HETE ([M^−^] = 319.2, peak 3). B) The conversion of [1^−14^C] AA into unidentified polar compounds (UPC) (peak 1) and α-ketol (peak 2) in response to wounding. CPM - counts per minute.

In *C. imbricata,* AA is an abundant polyunsaturated fatty acid, exceeding 20% of total fatty acids (unpublished data). The presence of endogenous eicosanoids of the AOS-LOX pathway, 8-HETE ([M^−^] = 319.2), α-ketol ([M^−^] = 335.2) and cyclopentenone ([M^−^] = 317.2) in *C. imbricata* was confirmed by RP-HPLC/MSMS analysis (Fig. 2A, EIC). The identification of the compounds was based on identical retention times and mass-spectra with *G. fruticosa* AOS-LOX products as standards [32].

### Arachidonic Acid Metabolism in Response to Wounding

The metabolites of AA formed by *C. imbricata* were detected at the time of wounding and in response to incision (Fig. 1). The formation of 8-HETE, α-ketol, and cyclopentenone during the incubation of the coral tissue homogenate with radiolabeled AA confirmed the catalytic activity of AOS-LOX (Fig. 2A, peaks 2 and 3). In the presence of the reducing agent SnCl_2_, the AOS reaction was avoided and the reduced intermediate 8-HETE was detected as the main product (data not shown). Besides the known products of the AOS-LOX pathway, the formation of additional compounds was detected (Fig. 2A, peak 1). As identification of these metabolites was out of the scope of the current research, two peaks visible on the radio chromatogram were not separated and the radioactivity was summarized. In the selected time frame, the level of α-ketol (Fig. 2A, peak 2) increased in response to wounding (in total 27%, Fig. 2B); at the same time the amount of unidentified polar compounds (UPC) (Fig. 2, peak 1) decreased equally (Fig. 2B).

### Sequence Analysis of *AOS-LOX*

The cloning and sequencing of *C. imbricata AOS-LOX* cDNAs resulted in two complete *AOS-LOX* sequences of encoding ORFs, along with the 5′- and 3′-UTRs, designated as *AOS-LOXa* (NCBI ID: KF000373) and *AOS-LOXb* (NCBI ID: KF000374). BLASTp analysis revealed high sequence homology with other cnidarian *AOS-LOX*s. The identity to other coral *AOS-LOX*s *(G. fruticosa* GenBank *accession number* EU082210.1; *Clavularia viridis* AB188528.1; *Plexaura homomalla* AF003692.1) was between 81–87%. The sequence identity between *C. imbricata AOS-LOXa* and *b* (88%) was lower than between the corresponding *G. fruticosa* paralogs (98%, personal data).

Based on the sequence analysis of the coral AOS-LOX fusion proteins, all catalytically important amino acids of LOX domains are conserved. In the primary structure of the LOX domain of AOS-LOXa and AOS-LOXb, the active site residue equivalent to that reported to be determinant of *R* or *S* stereospecificity of LOXs (glycine in *R*- and alanine in *S-*specific LOXs) [48] is G800 and G801, respectively. Thus the LOX domains of AOS-LOX fusion proteins convert the substrate fatty acid (AA) into 8*R-*HpETE.

Except for a conservative L150F substitution in AOS-LOXb, all catalytically important amino acids (H67, N147, L150; R345, Y349) of the AOS domains of *C. imbricata* are conserved. On the other hand, in the substrate binding pocket of the AOS domain of AOS-LOXb, the substitution of phenyl alanine 150 (as in catalase) instead of leucine (as in other coral AOS-LOX fusion proteins) and additional amino acid substitutions (K60E; F90Y, V156S; L176/177S), including one amino acid insertion (S161), were detected.

**Figure 3:**
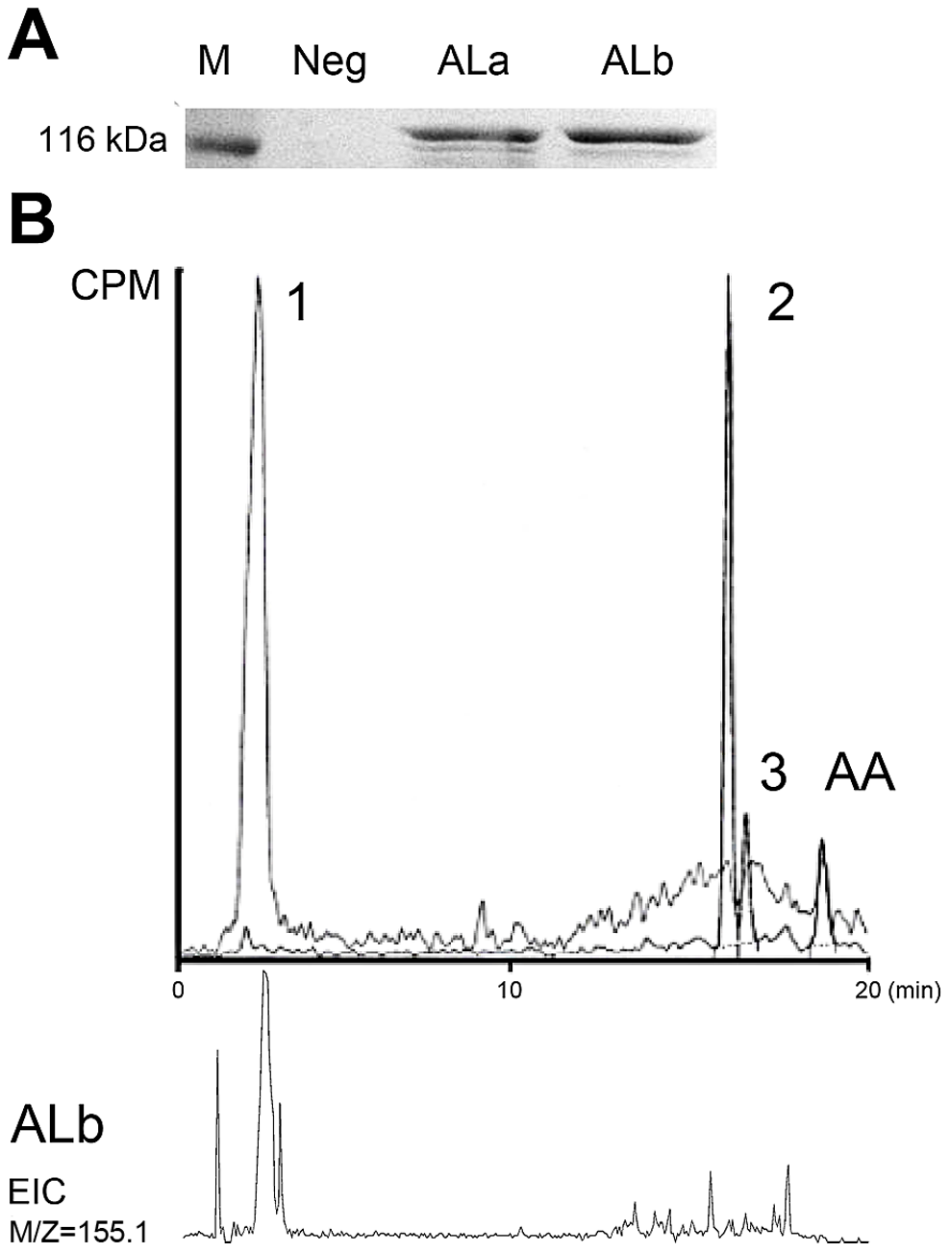
RP-HPLC analysis of incubation products of *C. imbricata* AOS-LOX fusion proteins expressed in *E. coli*. Bacterial extracts expressing the fusion proteins. M, protein molecular weight marker (Fermentas); Neg, negative control, pET11a vector without insert; ALa, AOS-LOXa; ALb, AOS-LOXb. The RP-HPLC analysis of the conversion of [1^−14^C] AA by AOS-LOXa (peaks 2, 3) and AOS-LOXb (peak 1). The peak numbers indicate identical compounds formed by the coral homogenate (Fig. 2A, and corresponding EIC) and expressed AOS-LOX proteins. EIC [M^−^] = 155.1 corresponding to the main AOS-LOXb product (peak 1). CPM - counts per minute.

To confirm their catalytic activity, the ORFs of the *AOS-LOX* fusion proteins were expressed in bacterial expression system, resulting in fusion proteins with expected size, 122.0 kDa for AOS-LOXa and 122.3 kDa for AOS-LOXb (Fig. 3, lanes ALa and ALb, respectively). The catalytic activity of AOS-LOXa was recovered in the 13000×g supernatant of sonicated cells with [1-^14^C] AA as the substrate. The formation of α-ketol (70% of total radioactivity) and cyclopentenone (14%) by AOS-LOXa was confirmed by RP-HPLC (Fig. 3, peaks 2 and 3, respectively). Accordingly, the AOS-LOXa product profile is identical to that of previously characterized fusion proteins [28], [32]. The main products formed from AA by AOS-LOXb (90% of total radioactivity) (Fig. 3, peak 1) co-eluted with the UPC (Fig. 2, peak 1). In the presence of mild reducing agent the LOX domains of both fusion proteins converted AA exclusively into 8-HETE. As incubation of AOS-LOXb with 8*R*- HpETE resulted in identical product profile compared to incubations with AA (data not shown), the novel activity can be attributed to the AOS domain of AOS-LOXb. Based on chromatographic behavior and mass-spectral data of AOS-LOXb products, we suggest the formation of an oxo-octenoic acid ([M^−^] = 155.1) from 8-HpETE.

Accordingly, both coral *AOS-LOX* genes encode for functional fusion proteins. While AOS-LOXa is the main source of the known compounds of AOS-LOX pathway, AOS-LOXb exhibits a novel activity. The identification of the products of AOS-LOXb is a matter of future research.

### Expression Analysis of AOS-LOX

Coral *C. imbricata* is also known by the common name Kenya tree coral because of its tree-like appearance. The normal expression levels of *AOS-LOXa* and *AOS-LOXb* were estimated in the “stem” and “branch” of the coral colony. Under normal conditions, higher expression levels of both transcripts were detected in stem tissue, while *AOS-LOXb* transcript was 2.8 times more abundant than *AOS-LOXa* (*P<*0.05). Consequently, all samples in one experimental set were taken from the same location (either stem or branch).

#### Repeated wounding (one colony)

**Figure 4:**
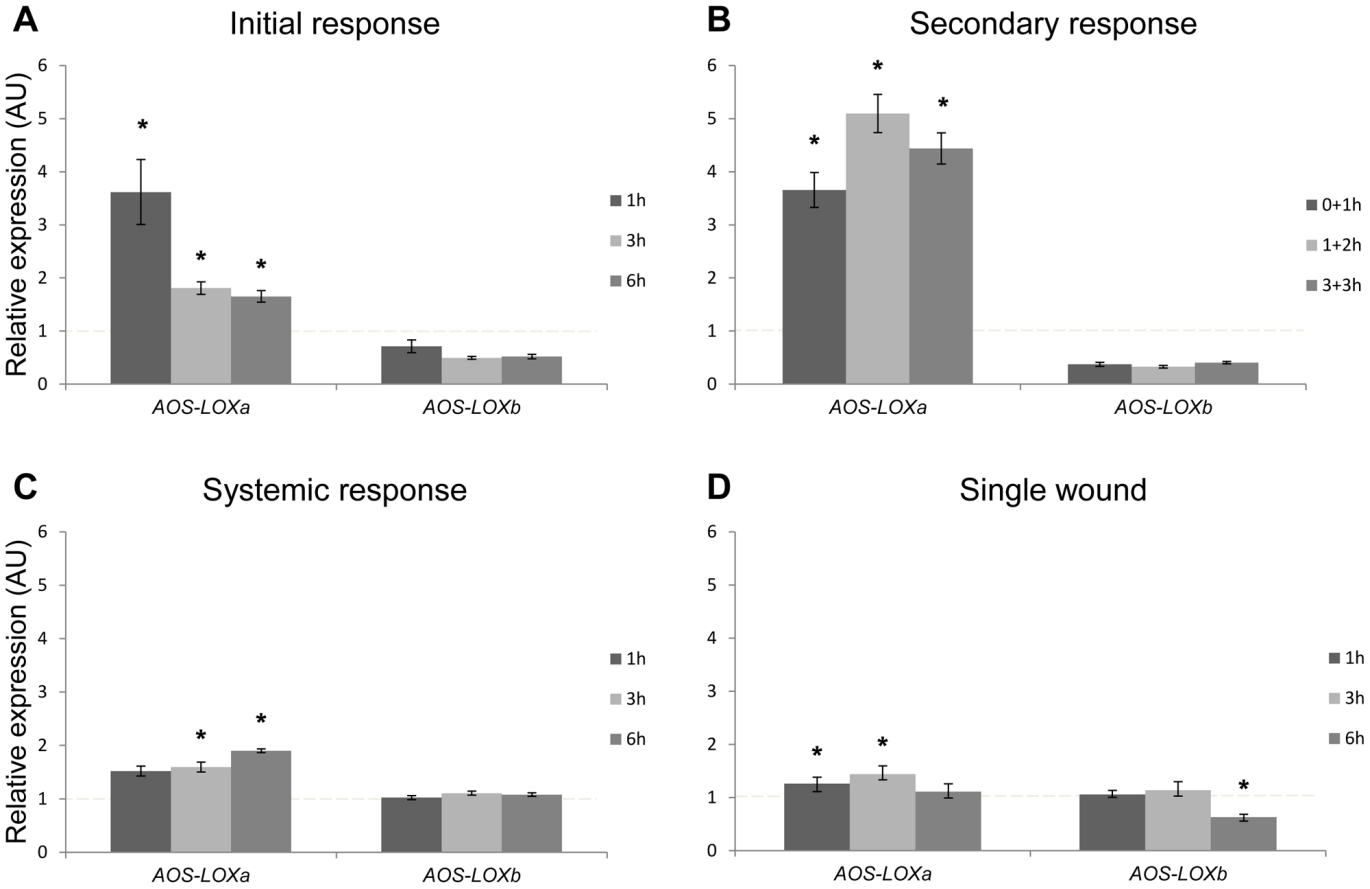
Quantitative real-time PCR analysis of transcript levels of *C. imbricata AOS-LOXa* and *AOS-LOXb*. Changes in gene expression in response to wounding within one colony: A) accumulative response; B) secondary response; C) systemic response. D) Response to a single wound. Data are means ± standard error, asterisk indicates significantly higher or lower expression relative to control (*P*<0.05).

To exclude genetic variation between coral colonies, the expression levels of *AOS-LOXa* and *AOS-LOXb* in response to incision wounding were estimated within a single octocoral colony. Significant up-regulation of *AOS-LOXa* was confirmed adjacent to the wounding site. The maximum fold change 3.6 was recorded at one hour post wounding, decreasing at 3 h and 6 h to 1.8- and 1.6-fold, respectively (*P<*0.05) (Fig. 4A).

To assess the secondary response, the transcript levels were recorded in tissue adjacent to the wounding site at (0+1 h), (1 h+2 h) and (3 h+3 h). The fold change of *AOS-LOXa* at (0+1 h) was identical to the 1 h sample, indicating that a similar wound response was generated near the wounding site. An additional increase was detected in the (1 h+2 h) and (3 h+3 h) samples, with up-regulation of 5.0- and 4.4-fold (*P<*0.05), respectively (Fig. 4B). The data indicate that the severity of stress had a direct effect on *AOS-LOXa* transcription.

In a parallel experiment, the occurrence of a systemic response was investigated. The transcript level was recorded in distal parts of the coral colony at the tips of the branches. Although not reaching as high level as detected around the wounding site, *AOS-LOXa* was significantly up-regulated at 3 h and 6 h post wounding, by 1.5- and 1.9-fold (*P<*0.05), respectively. At the same time, the *AOS-LOXb* expression remained constant (Fig. 4C). The up-regulation of *AOS-LOXa* at distal parts of the colony indicated enhanced alertness of the whole colony.

It should be noted that after repeated wounding the coral was visibly stressed. After incision, all polyps of the colony instantly closed and remained closed while the area close to the wound contracted. Repeated wounding induced the color changes in the remaining colony. However, all treated colonies were able to recover and regenerate between four and ten days, depending on the severity of the injury.

#### Single wounding (separate colonies)

In order to detect and compare the response between repeated and single incision wounding, the expression levels of *AOS-LOXa* and *AOS-LOXb* were estimated in separate coral colonies having identical genetic background. Adjacent to the wounding site, *AOS-LOXa* was significantly up-regulated at 1 h and 3 h post wounding by 1.2- and 1.4-fold (*P<*0.05), reaching the normal level at 6 h (Fig. 4D), while *AOS-LOXb* was significantly down-regulated only at 6 h with a relative expression ratio of 0.6 (*P<*0.05) (Fig. 4D). The moderate up-regulation of *AOS-LOXa* in response to a single wound also supports the contention that stress severity has an impact on *AOS-LOXa* expression.

## Discussion

Using an acute incision wounding model, we showed that in response to wounding the soft coral *C. imbricata* undergoes rapid up-regulation of the AOS-LOX pathway *in vivo*. A major implication of our results is that the eicosanoid pathways used by coral during tissue injury and healing are similar to the oxylipin pathways in plant wound responses, highlighting a conserved pathway throughout the animal and plant kingdoms. The results of the current study establish coral AOS-LOX route as rapid-onset stress response pathway. Two *AOS-LOX* genes were found to be differentially responsive to mechanical injury, with *AOS-LOXa* transiently up-regulated (peaking at one hour post injury) while *AOS-LOXb* expression remained stable. The differential expression regulation and competition for the same upstream substrate (AA) implicate their distinct biological function in the wound response.

The initial wound response in animals, including corals, aims for rapid and efficient provisional plugging of the wound to minimize both the loss of vital fluids and environmental challenges (e.g. bacterial contamination) [10], [49]–[51]. In vertebrate animals, the immediate release of cell-derived damage signals, including Ca^2+^, ATP and reactive oxygen species (ROS), defines the wound area and severity of damage within the first minutes post injury [52]. Acute-onset signals initiate the secondary phases of wound healing through the transcription of wound response genes, including 1) immediate wave: genes expressed within 30 min to 1 h, the expression of which ceases within 6–12 h and in which protein synthesis is not required prior to induction, 2) fast phase: expression lasting from 30 min up to 12 h, and 3) sustained wave: lasting from 3 to 12 h [53]. Thus the time course of *AOS-LOXa* expression in *C. imbricata* is consistent with the immediate wave pattern.

Because of their sessile nature, corals share many plant-like physiological features. Likewise, the *AOS-LOXa* expression pattern in *C. imbricata* shows homology to lipoxygenase pathway expression in plant wound response. The wound response in plants includes the instant release of Ca^2+^, ROS and leaf volatiles (e.g. short chain aldehydes) and traumatin [54], [55], accompanied by the rapid synthesis and accumulation of the stress hormone JA via a lipoxygenase pathway, involving LOX, AOS, AOC steps [56], [57]. To induce the synthesis of JA only one of four *Arabidopsis thaliana* 13(*S*)-LOXs (mainly found in plant plastids) is up-regulated an hour upon wounding [58]–[60]. Matching the same time window, the sequential step catalyzed in plastids by AOS is also up-regulated at the transcriptional [61], [62] and translational level [24]. Thus, although the downstream mediators may diverge, the immediate wave expression of LOX and AOS after wounding is a shared, conserved pathway preparing the transit from provisional danger signaling to the initiation of structurally consolidating tissue repair. Whereas in plants the expression of *LOX* and *AOS* is regulated separately, in coral the AOS-LOX fusion protein enforces strict stoichiometric coupling of both catalytic steps.

The divergent product profile generated in the coral can be explained using plant oxylipin pathways as a guide. In plants the fatty acid hydroperoxide generated from α-linolenic acid by 13-LOX is converted in parallel by AOS [61], [63], [64] and hydroperoxide lyase (HPL) [65], [66]. After wounding the AOS route leads to the formation of JA [61], [63], while volatile C_6_ aldehydes (e.g. Z-3-hexenal or E-2-hexenal) and non-volatile oxylipins (e.g. traumatin) are instantly generated by HPL [62], [64], [65], [67]. Analogously to the reactions of plant LOX and AOS enzymes, the coral AOS-LOXa fusion protein catalyzes the formation of labile allene epoxide, although using AA instead of α-linolenic acid as a substrate.

Hypothetically, if the HPL-like reaction using 8-HpETE formed from AA by the 8-LOX domain of the AOS-LOX fusion protein takes place in the coral, two compounds with masses of 156 (C1–C8) and 180 (C9–C20) can be formed. The radiolabeled AA metabolite formed *in vitro* by *C. imbricata* AOS-LOXb has a mass of 156, which corresponds to (C1–C8) and also matches the location of [1-^14^C] label, therefore suggesting that the HPL-like reaction has indeed taken place in coral by AOS-LOXb. The existence of parallel AOS-LOXa and AOX-LOXb pathways suggest that the coral initial wound response could also include the instant formation of aldehydes and oxo-acids.

As mentioned earlier, after wounding the concurrent conversion of the same substrate (AA) by *C. imbricata* (Fig. 2B) resulted in the gradual enhancement of α-ketol (Fig. 2A, peak 2) corresponding to AOS-LOXa and a decrease in AOS-LOXb metabolites (Fig. 2A, peak 1). The shift in metabolite spectrum after wounding is best explained by an altered balance between AOS-LOXa and AOS-LOXb, resulting in a dominance of AOS-LOXa products. A sustained increase in enzyme activity may be reached by up-regulation of gene expression and *de novo* protein synthesis or through the activation of AOS-LOX by increased cellular Ca^2+^, shown to enhance the activity of *P. homomalla* and *G. fruticosa* AOS-LOXs *in vitro* [32], [68]. Further studies are needed to identify all *in vivo* metabolites, as well as to specify the role and regulation of AOS-LOX isoforms in coral stress.

In conclusion, although plants and corals use different fatty acid precursors, the reactions and time course of oxylipin formation via LOX and AOS pathways in response to wounding are similar, suggesting conserved signaling pathways.
